# Transesophageal Echocardiography Guidance to Prevent and Manage Pitfalls from Abdominal Normothermic Regional Perfusion and Optimize Timing during Organ Retrieval from a Donor after Circulatory Death

**DOI:** 10.3390/jpm13071177

**Published:** 2023-07-24

**Authors:** Amedeo Bianchini, Cristiana Laici, Noemi Miglionico, Maria Giacinta Bianchi, Elena Tarozzi, Enrico Bernardi, Jessica Toni, Erika Cordella, Giovanni Vitale, Antonio Siniscalchi

**Affiliations:** 1 Post-Surgical and Transplant Intensive Care Unit, IRCCS Azienda Ospedaliero-Universitaria di Bologna, 40138 Bologna, Italy; amedeo.bianchini@aosp.bo.it (A.B.); cristiana.laici@aosp.bo.it (C.L.); enrico.berardi@aosp.bo.it (E.B.); jessica.toni@aosp.bo.it (J.T.); antonio.siniscalchi@aosp.bo.it (A.S.); 2 Department of Medical and Surgical Sciences (DIMEC), University of Bologna, 40126 Bologna, Italy; noemi.miglionico@studio.unibo.it (N.M.); maria.bianchi7@studio.unibo.it (M.G.B.); elena.tarozzi@studio.unibo.it (E.T.); 3 Emilia-Romagna Transplant Reference Centre, IRCCS Azienda Ospedaliero-Università di Bologna, 40138 Bologna, Italy; erika.cordella@aosp.bo.it; 4 Internal Medicine Unit for the Treatment of Severe Organ Failure, IRCCS Azienda Ospedaliero-Universitaria di Bologna, 40138 Bologna, Italy

**Keywords:** donation after circulatory death, extracorporeal membrane oxygenation, liver transplantation, normothermic regional perfusion, transesophageal echocardiography

## Abstract

An essential means of collecting more abdominal donor organs is controlled donation after circulatory death (cDCD). The organs are typically preserved during cDCD using the abdominal normothermic regional perfusion (A-NRP) technique to recirculate oxygenated blood flow following cardiac arrest and the withdrawal of life support. One of the challenges of A-NRP is ensuring the correct vascular devices’ positionings, specifically extracorporeal membrane oxygenation cannulae and aortic balloons, typically achieved through fluoroscopy with or without contrast agents. Here, we present a case report in which transesophageal echocardiography (TEE) helped the transplant team to effectively procure viable abdominal organs from a cDCD donor in the shortest time frame, as minimizing time is one of the most crucial factors in maintaining organ viability. TEE use leads to a more effective and efficient A-NRP procedure with limited complications. In addition, it allows us to observe the circulation of both the thoracic and part of the abdominal organs using one fast exam. This case is the first report describing TEE as a primary guide and useful tool for DCD donors. However, prospective studies are needed to confirm that TEE could be used as standard practice during all DCD organ retrieval procedures.

## 1. Introduction

Thousands of patients die each year due to a lack of available donor organs, and those with complications or underlying conditions are often precluded from even being eligible for the donor waitlist. An essential means of collecting more abdominal donor organs is controlled donation after circulatory death (cDCD). The organs are typically sustained during cDCD using the normothermic regional perfusion (NRP) technique to recirculate oxygenated blood flow following cardiac arrest and the withdrawal of life support. 

One of the challenges of NRP is to ensure the correct guidewire positionings, specifically cannulae and aortic balloons (ABs), which is typically achieved through fluoroscopy with or without contrast agents, radiography, and transesophageal echocardiography (TEE). Already considered an excellent technique for cardiac and endovascular procedure guidance, cardiac disease assessment, and hemodynamics monitoring [1], TEE provides a clear image of the mediastinal structures without air or rib interference. Some authors have also suggested TEE use in evaluating pulmonary diseases and liver blood flow, such as when assessing hepatic venous drainage and portocaval anastomosis integrity for liver transplantation [LT] [2,3,4,5]. Furthermore, TEE is commonly used for ultrasound (US)-guided extracorporeal membrane oxygenation (ECMO) cannula placement [6] and to verify infra-AB position [7,8] in cardiogenic shock or during cardiovascular surgery.

However, the potential role of TEE to guide abdominal DCD organ donation is less commonly described. Here, we present a case report in which TEE helped the transplant team to effectively procure viable abdominal organs from a DCD donor in the shortest time frame, as minimizing time is one of the most crucial factors in maintaining organ viability. This issue is especially relevant in Italy, where national guidelines for confirmation of death require a twenty-minute wait to check asystole on continuous ECG monitoring before the transplant team can begin organ retrieval. 

The non-invasive nature of TEE, its accessibility, its adaptability to different positions and spaces, and the safety of its use for both the technicians and the health of the organs outweigh any potential disadvantages. Furthermore, using TEE as a guide can lead to a more effective and efficient NRP procedure with limited complications. It allows us to observe the circulation of the thoracic and part of the abdominal organs using one fast exam. 

We present this case study as a model highlighting how TEE can be routinely used during DCD organ recovery. This report is the first case describing TEE as a primary guide and essential tool for DCD donors, so that TEE could be adopted as standard practice during all DCD organ retrieval procedures.

## 2. Materials and Methods

We described the method used by the hospital’s abdominal NRP (A-NRP) team in one case of TEE-guided DCD donation performed on 1 November 2022, at IRCCS Azienda Ospedaliero-Universitaria di Bologna, Italy. A GE LOGIQ 7 ultrasound machine with a GE 6T transesophageal probe was used by a trained anesthetist. 

As a circulatory and pulmonary support system for NRP, we employed the Maquet Cardiohelp System while, for the venous and arterial NRP cannulae, we used the Medtronic Biomedicus model.

In the cDCD donation process, national guidelines consider specific consent for the maneuvers and treatments carried out before the ascertainment of death unnecessary, so long as these are aimed at preparing the donor for organ donations about which the family has been given clear and complete information. Consent to donate organs is given either expressly and directly by the donor in life or, if the donor did not express their will on the matter during life, the family is informed of the issue and, if no entitled family members express their opposition, organs may be collected according to the cDCD process [9]. 

This study was conducted following ethical guidelines of the World Medical Association’s Declaration of Helsinki and guidelines for Good Clinical Practice [10,11]. Finally, we have attached more explanatory ultrasound images of the case.

## 3. Case Presentation

A 70-year-old male former smoker was sent to S. Orsola-Malpighi Hospital in Bologna after suffering cardiac arrest at home. The man already had a pacemaker (PM) previously inserted due to carotid atheroma disease and sinus node dysfunction. Advanced Cardiovascular Life Support maneuvers were immediately performed upon reaching the hospital, and spontaneous circulation returned after 20 min. He was then admitted to the intensive care unit (ICU) in a post-anoxic coma, where invasive ventilation was needed. Hospitalization was complicated by a persistent coma, refractory epilepsy, and a pulmonary infection that led to septic shock. Repeated neurological evaluations and radiological investigations showed irreversible brain damage, making the patient a candidate for therapeutic withdrawal.

Usually, the definition of a brain-dead patient with an irreversible loss of neurological functioning follows national guidelines. Subsequently, the patient may be a candidate for a possible DBD donation (donation after brain death). 

In cases where the criteria are not met for neurological factors of death, it is possible to decide with the entitled family members to limit the intensive treatments. Upon discontinuation of treatment, the patient goes into cardiac arrest. The absence of circulation for a prolonged time causes irreversible brain damage with the death of the brain tissue. In Italy, brain damage is considered irreversible after 20 min of cardiac arrest (the no-touch period), and a death diagnosis can be made, after which oxygenated blood flow to the organs can be restored through NRP to perform a cDCD donation [9]. The evaluation of contraindications for the cDCD donation, including an analysis of the risk of donor-derived infections, occurs through an interdisciplinary assessment that includes consultation with the local referral transplant committee.

During cDCD preparation, we maintained mean arterial pressure (MAP) above 65 mmHg with norepinephrine. Following hospital protocol, vascular introducers were placed in the femoral venous and arterial vessels ante-mortem, and heparin was administered during the agonal period.

Before therapeutic withdrawal, we inserted guidewires and an aortic occlusion catheter using transesophageal US guidance as prescribed and permitted by national law [12]. 

**Figure 1:**
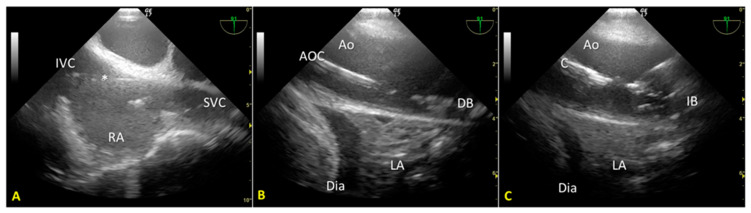
(**A**). Mid-esophageal bicaval view. The guidewire (*) was inserted through the inferior vena cava into the right atrium. (**B**). Mid-esophageal descending aorta long-axis view. The aortic occlusion catheter is visualized in the thoracic aorta; the deflated balloon is located just above the diaphragm. (**C**). Mid-esophageal descending aorta long-axis view during A-NRP. The position of the AB (inflated) remained unchanged above the diaphragm. IVC—inferior vena cava; RA—right atrium; SVC—superior vena cava; * guidewire; Ao—aorta; AOC—aortic occlusion catheter; LA—lung atelectasis; Dia – diaphragm; DB—desufflated balloon; IB—inflated balloon.

We used an introducer needle to insert a Super Stiff guidewire into the right femoral vein until the right atrium. Correct positioning of the guidewire was verified in real time using the TEE mid-esophageal bicaval view (Figure 1A). Next, we inserted a second guidewire through the right femoral artery sheath and introduced it up to the thoracic aorta. After visualizing the wire’s tip within the thoracic aorta using the mid-esophageal descending aorta short-axis view, we partially withdrew the guidewire so that its tip stopped below the diaphragm. These two guides allowed the introduction of venous and arterial A-NRP cannulae post-mortem. 

Finally, we introduced a third guidewire through the left femoral artery sheath to allow proper stent placement for aortic occlusion. TEE use guided and monitored correct positioning using the mid-esophageal descending aorta long-axis view (Figure 1B,C). Using fluoroscopy, we double-checked the proper positioning of the guides and aortic occlusion catheter (AOC).

**Figure 2:**
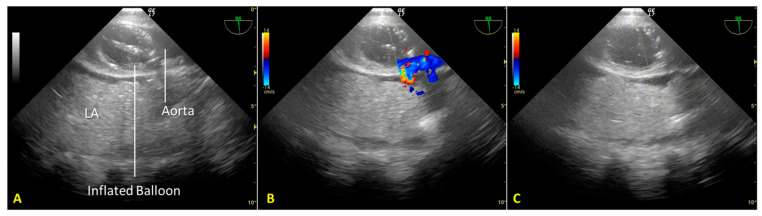
(**A**). Mid-esophageal descending aortic long-axis view scan: the endovascular occluder (RELIANT^®^ catheter balloon, Tamworth, UK) is visible in the descending thoracic aorta. (**B**). Incomplete occlusion of the thoracic aorta during the initial phase of regional extracorporeal support for organ retrieval. Transesophageal color Doppler echocardiography documents the passage of blood around the aortic occluder. (**C**). Complete occlusion of the thoracic aorta after additional filling of the RELIANT^®^ AB. Absence of blood flow in the thoracic aorta above the occluder. LA—lung atelectasis.

Cardiac arrest occurred rapidly with the cessation of life support. After checking the asystole on continuous ECG monitoring for twenty minutes (“no-touch period”), we certified the patient’s death. We performed post-mortem percutaneous femoral cannulation using the previously placed introducer wires. A 19 Fr cannula was placed in the right femoral vein (draining cannula) and a 17 Fr cannula in the right femoral artery (reinfusion cannula). Before starting the A-NRP, we inflated the AB according to the manufacturer’s instructions, which—considering the thoracic aorta diameter of 30 mm, as measured from a recent CT exam—was with 19 mL of saline (Figure 2A). We set extracorporeal blood flow to be greater than 2.4 L/min/m^2^, corresponding to half of the patient’s body surface area.

Nevertheless, abdominal aortic regional pressure remained consistently suboptimal (approximately 45 mmHg) [12]. A transesophageal color Doppler echocardiography scan to obtain a mid-esophageal descending aortic long-axis view demonstrated incomplete occlusion of the thoracic aorta (Figure 2B). Due to atheromatous plaques, the incomplete aortic occlusion required an additional 15 mL of saline. Real-time US-guided insufflation made filling the aortic balloon (AB) possible. AB is just enough to achieve total aortic occlusion but not so much as to risk balloon hyperinflation, which could cause aortic rupture, balloon rupture, and abdominal AB extension with celiac trunk occlusion (Figure 2C).

**Figure 3:**
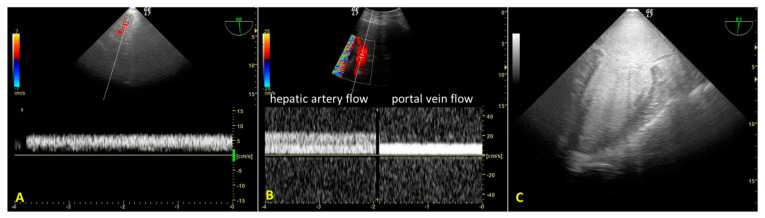
(**A**). Transesophageal color Doppler echocardiography with transgastric approach during the initial phase of regional extracorporeal support. Slow hepatic artery flow is sampled due to incomplete aortic occlusion (continuous right hepatic artery flow velocity = 7 cm/s). Portal vein flow was absent. (**B**). Color Doppler ultrasound with convex probe after complete aortic occlusion. Right hepatic artery flow was increased (velocity 25 cm/s) and the portal flow was sampled (velocity 12 cm/s). (**C**). Absence of flow in the left ventricle and atrium after complete aortic occlusion (mid-esophageal two-chamber view).

During A-NRP, we monitored hepatic re-perfusion with color Doppler US and laboratory tests. TEE revealed initial reduced hepatic artery blood flow (transgastric view) due to incomplete aortic occlusion and decreased hepatic perfusion pressure (Figure 3A). After complete aortic occlusion, hepatic artery blood velocity increased, and portal vein blood flow was sampleable. (Figure 3B) At the same time, blood lactates decreased from 9.4 mEq/L to 5.2 mEq/L. TEE also allowed us to observe the absence of blood flow in the left ventricle and atrium after complete aortic occlusion, as well as the lack of cardiac contractility, despite PM activity displayed on the electrocardiogram (Figure 3C).

Once cDCD preparations were complete, the patient underwent abdominal organ removal. Unfortunately, the kidneys were ineligible for transplantation due to a high biopsy score, while the LT was performed without complications.

**Figure 4:**
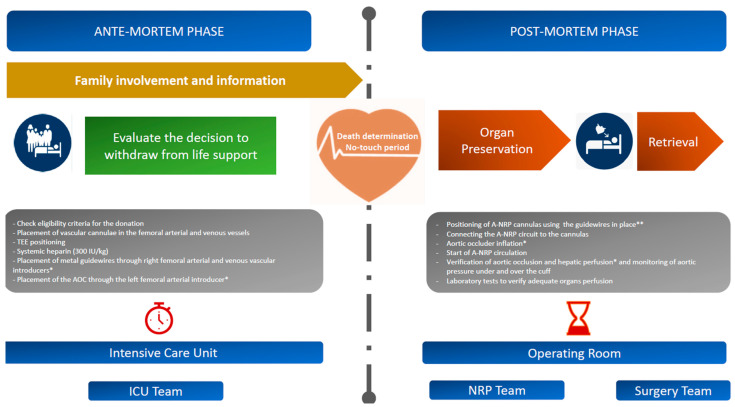
Visual timeline of the events in subjects undergoing the DCD process at IRCCS Azienda Ospedaliero-Universitaria di Bologna. A-NRP—abdominal normothermic regional perfusion; AOC—aortic occlusion catheter; ICU—intensive care unit; IU—international units; Kg—kilograms; NRP—normothermic regional perfusion; TEE—transesophageal echocardiography. * TEE echocardiographic guide is used to verify correct placement. ** According to the direct Seldinger technique.

Figure 4 depicts a visual chronology of the events in the DCD process at the IRCCS Azienda Ospedaliero-Universitaria of Bologna, the locations where they occur, and the professional figures involved. Furthermore, it shows the procedures in which the TEE is used as a diagnostic and interventional support.

In summary, the timeline of the phases associated with the DCD was as follows:

- Ischemia time, warm 53 min;
- Ischemic functional time (interval from MAP < 50 mmHg to NRP start time), 46 min;
- Time from the withdrawal of ACC treatment, 29 min;
- Interval time from cardiac arrest to NRP start time, 26 min (20 min for “no-touch” period and 6 min for cannulation insertion and regional perfusion);
- Aortic balloon inflation takes place immediately before starting NRP.

Regarding the clinical course after LT, the last follow-up was dated 26 May 2023, with good clinical conditions and graft function. Immunosuppressive therapy was tacrolimus. Finally, we observed no opportunistic infections, rejections, or impaired kidney function during the follow-up period.

## 4. Discussion

**Figure 5:**
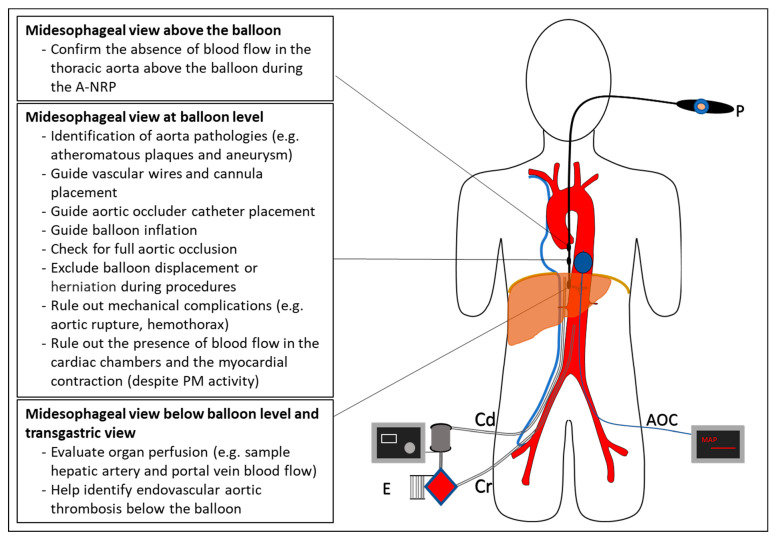
Role of TEE to guide abdominal DCD organ donation. E—extracorporeal membrane oxygenator; Cd—drainage cannula; Cr—re-infusion cannula; AOC—aortic occlusion catheter; M—pressure monitors; P—TEE probe.

A-NRP maintains allografts during DCD with extracorporeal blood circulation, which allows for the support of abdominal organ perfusion and recovery from warm ischemic injury post-mortem. In A-NRP, the circuit consists of a VA extracorporeal circuit (i.e., venous drainage cannula size 21/23 Fr, arterial flow cannula size 17/19 Fr connecting tubing, and a centrifugal pump (Figure 5) that permits blood to drain from the venous circuit through the inferior vena cava, become oxygenated extracorporeally, and then return to the abdominal aorta. In addition, a balloon catheter is inserted through the abdominal aorta and clamped just the diaphragm, thus preventing blood from escaping into the thoracic organs and brain. 

While highly effective when performed correctly, this technique poses several potential pitfalls that we must anticipate in order to ensure successful transplantation. First, brain perfusion must be excluded appropriately; otherwise, using A-NRP invalidates the death pronouncement because the permanent cessation of blood flow towards the brain determines death. Secondly, the transplant team must not delay percutaneous cannula placement due to difficulties such as vessel perforation or cannula dislocation; otherwise, it could prolong the recovery time from warm ischemic injury. Lastly, perfusion must be optimally between 2 L/min and 2.5 L/min, and we must maintain the blood pressure at 60–65 mmHg; otherwise, the abdominal organ could suffer irreversible damage.

However, when performed correctly, A-NRP ensures the continuous perfusion of warm blood, which consequently sustains organ viability for DCD, namely by restoring abdominal organ function, mitigating organ injury, promoting energy storage, maintaining homeostasis, reducing the time spent by the organ in warm ischemia, and allowing the organs to recover by establishing organ perfusion. Furthermore, the technique permits us to assess the viability of abdominal organs in a non-ischemic state (instead of from direct cold storage) before retrieval. 

NRP has several advantages over other techniques for DCD organ retrieval. First, it minimizes the need for multiple ex situ perfusion devices for each organ, which is highly costly and less efficient. Secondly, NRP may reduce the risk of liver cholangiopathy when performed before recovery, as shown in recent studies by Watson CJ et al. [13].

For these reasons, many transplant centers are moving away from immediate cold preservation and recovery following death and relying instead on NRP to temporarily restore oxygenated blood flow to abdominal and, more recently, thoracic organs before recovery [14,15,16].

TEE helps resolve potential NRP-related issues during DCD organ harvesting by optimizing best practices in guidewire and cannula placement, aortic occlusion, organ blood flow, and all other aspects of the DCD organ retrieval procedure.

In our case study, TEE helped us properly insert the guidewires, A-NRP cannulae, and AB catheter to verify the correct positioning of the AB throughout the procedure, to guide in real-time AB inflation, and to verify the aortic occlusion during A-NRP. Furthermore, TEE allowed us to rule out the presence of blood flow in the cardiac chambers and the presence of cardiac contraction, despite PM activity. 

Here, TEE helped us to determine the optimal AB inflation (35 mL), which prevented the re-perfusion of the brain and the heart and, therefore, a potential breaking of the donation procedure (e.g., due to cardiac electrical activity recovery). On the other hand, TEE has helped us to avoid balloon overstretching (which increases the risk of aortic rupture and massive bleeding) or balloon herniation up to the celiac trunk, causing liver ischemia.

Fluoroscopy with contrast agents offers another method of verifying the correct positions of the vascular devices and total aortic occlusion during A-NRP. However, fluoroscopy has notable limitations. For example, it may not always be available, it requires a dedicated technician, it is cumbersome, it can limit DCD procedures, it is often incompatible with the ICU bed, it exposes healthcare personnel to radiation and the kidneys to toxicity from contrast agents, and finally, it cannot identify AB hyperinflation. Furthermore TEE better verifies the positioning of the intra-AB pump (IABP) and is a superior technique to radiography and other methods [8]. 

In our case study, TEE and percutaneous abdominal US helped verify the efficiency of A-NRP by assessing abdominal organ perfusion. The reduced blood flow velocity in the hepatic artery and the inability to sample the portal vein blood flow (as well as a reduced MAP) indicated hepatic hypoperfusion. Using TEE, we looked for the cause of the hypoperfusion, identified it as incomplete aortic occlusion, and we corrected it (unfortunately, there is currently no standard limit for blood flow velocity during A-NRP and more studies are needed to verify the impact of mean blood flow velocity on recovery of organ function).

Based on these experiences and considerations, our A-NRP team has adopted TEE to guide DCD procedures. In addition, the clinical case suggests using TEE to also identify other causes of abdominal organs’ hypoperfusion and to pursue specific therapies to correct them. For example, hepatic hypoperfusion due to AB caudal displacement with occlusion of the celiac trunk (e.g., chest compression maneuvers performed on donor in case of prolonged time to initiation of A-NRP) can be recognized through TEE and then corrected in real-time ultrasound vision. In other instances, TEE can help identify endovascular aortic thrombosis, for which we can administer additional heparin. On the other hand, TEE can help identify significant bleeding (e.g., aortic rupture or hemothorax) and guide large transfusions, consequently reducing the time to organ retrieval, which is crucial in maintaining the viability of the organs. This issue is critical in Italy, where organ retrieval cannot be initiated until 20 min after the pronouncement of death. 

Regarding this ethical issue, Italian legislation, unlike other countries, provides all possible guarantees [9]; the suspension of life-support treatments following cardiac arrest requires that the A-NRP circuit starts only after the clinical diagnosis of death. It is carried out without cardio-circulatory activity by checking the asystole on continuous ECG monitoring for 20 min. After confirmation of the death, we can use the A-NRP circuit to minimize the time of warm ischemia of organs.

As the perfusion to the brain determines death, the flow around the aortic balloon after the declaration of death may still pose an ethical dilemma.

About this point, the national guidelines [9] only prescribe monitoring of asystole during the “no-touch period” period. There is currently no guidance or requirement for imaging or blood flow monitoring. Excluding blood flow around the aortic balloon as observed through fluoroscopy or other imaging techniques (such as Trans Cranial Color Doppler sonography) is not required or specified. However, this is a complex ethical question that merits further discussion.

While relatively expensive, the notable advantages of TEE suggest that it should be made readily available as a part of transplant and donor center policy. TEE is a versatile tool with numerous benefits in DCD and other procedures and monitoring performed in the surgery room and the ICU, especially in cases where space is scarce (as fluoroscopy is much more cumbersome and requires more space) or the patient has a severe vascular disease with known or suspected aortic pathology.

TEE can play a fundamental role in all phases of the DCD donation procedure: guiding devices’ placement, verifying their proper positioning, and assessing the flow to avoid liver hypoperfusion. Therefore, it should always be available during a DCD donation.

The main limitation of this report derives from the absence of data comparing the use of TEE vs. non-TEE procedures in the NRP process to know which technique best improves the prognosis of the transplant patient receiving an organ during cDCD.

However, we do not have any prospective case studies in the literature comparing the impact of TEE application on short- and long-term outcomes in the recipient; our case, to the best of our knowledge, is the first report describing the use of the TEE in cDCD.

More data and prospective studies are needed to confirm that TEE could be applied as standard practice during all DCD organ retrieval procedures.

## Data Availability

Data supporting reported results can be found at IRCCS Azienda Ospedaliero–Universitaria di Bologna, Department of Digestive, Hepatic, and Endocrine–Metabolic Diseases, Post-Surgical and Transplant Intensive Care Unit.

## Abbreviations

AB	Aortic Balloon
A-NRP	Abdominal Normothermic Regional Perfusion
Ao	Aorta
AOC	Aortic Occlusion Catheter
Cd	Drainage Cannula
cDCD	Controlled Donation after Circulatory Death
Cr	Re-Infusion Cannula
DB	Desufflated Balloon
DBD	Donation after Brain Death
DCD	Donation after Circulatory Death
Dia	Diaphragm
E	Extracorporeal Membrane Oxygenator
ECMO	Extracorporeal Membrane Oxygenation
V-A ECMO	Veno-Arterial Extracorporeal Membrane Oxygenation
IABP	Intra-Aortic Balloon Pump
IB	Inflated Balloon
ICU	Intensive Care Unit
IVC	Inferior Vena Cava
LA	Lung Atelectasis
LT	Liver Transplantation
M	Pressure Monitors
MAP	Mean Arterial Pressure
NRP	Normothermic Regional Perfusion
P	TEE Probe
PM	Pacemaker
RA	Right Atrium
SVC	Superior Vena Cava
TEE	Transesophageal Echocardiography
US	Ultrasound
V-A	Veno-Arterial
